# Representações sociais de mulheres sobre cateterização venosa para procedimento anestésico-cirúrgico**

**DOI:** 10.15649/cuidarte.1258

**Published:** 2022-08-07

**Authors:** Herica Silva Dutra, Cristina Arreguy-Sena, Fernando Cordeiro Ribeiro, Luciene Muniz Braga, Paula Krempser, Laércio Deleon de Melo

**Affiliations:** 1 Universidade Federal de Juiz de Fora (UFJF), Faculdade de Enfermagem da UFJF (Facenf- UFJF), Programa de Pós- Graduação em Enfermagem. Juiz de Fora, Brasil. Autor de Correspondência. E-mail: herica.dutra@ufjf.br Autor de correspondência Universidade Federal de Juiz de Fora Universidade Federal de Juiz de Fora Faculdade de Enfermagem Juiz de Fora Brazil herica.dutra@ufjf.br; 2 Universidade Federal de Juiz de Fora (UFJF), Faculdade de Enfermagem da UFJF (Facenf- UFJF), Programa de Pós- Graduação em Enfermagem. Juiz de Fora, Brasil. E-mail: cristina. arreguy@ufjf.edu.br Universidade Federal de Juiz de Fora Universidade Federal de Juiz de Fora Faculdade de Enfermagem da UFJF Juiz de Fora Brazil cristina. arreguy@ufjf.edu.br; 3 Universidade Federal de Juiz de Fora (UFJF), Faculdade de Enfermagem da UFJF (Facenf- UFJF), Programa de Pós- Graduação em Enfermagem. Juiz de Fora, Brasil. E-mail: fernandoenfer@gmail. com Universidade Federal de Juiz de Fora Universidade Federal de Juiz de Fora Faculdade de Enfermagem Juiz de Fora Brazil fernandoenfer@gmail. com; 4 Universidade Federal de Viçosa (UFV), Departamento de Enfermagem e Medicina. Viçosa, MG - Brasil. E-mail: luciene.muniz@ufv.br Universidade Federal de Viçosa Universidade Federal de Viçosa Departamento de Enfermagem e Medicina Viçosa MG Brazil luciene.muniz@ufv.br; 5 Universidade Federal de Juiz de Fora (UFJF), Faculdade de Enfermagem da UFJF (Facenf- UFJF). Juiz de Fora, MG - Brasil. E-mail: paula@krempser.com.br Universidade Federal de Juiz de Fora Universidade Federal de Juiz de Fora Faculdade de Enfermagem Juiz de Fora MG Brazil paula@krempser.com.br; 6 Universidade Federal de Juiz de Fora (UFJF), Faculdade de Enfermagem da UFJF (Facenf- UFJF), Programa de Pós- Graduação em Enfermagem. Juiz de Fora, Brasil. E-mail: laerciodl28@hotmail.com Universidade Federal de Juiz de Fora Universidade Federal de Juiz de Fora Faculdade de Enfermagem Juiz de Fora Brazil laerciodl28@hotmail.com

**Keywords:** Saúde da Mulher, Cateterismo Periférico, Centros Cirúrgicos, Psicologia Social, Teoria de enfermagem, Women´s Health, Catheterization, Peripheral, Surgicenters, Psychology, Social, Nursing Theory, Salud de la Mujer, Cateterismo Periférico, Centros Quirúrgicos, Psicología Social, Teoría de Enfermería

## Abstract

**Introdução:**

Acateterizaçãovenosaéessencialparaprocedimentos anestésico-cirúrgicos, com características compatíveis com estabilidade, volume de fluxo em curtos espaços de tempo e grosso calibre a fim de evitar trauma vascular.

**Objetivo:**

Identificar as representações sociais de mulheres sobre punção venosa para fins anestésico-cirúrgicos à luz dos estressores de Neuman.

**Materiais e métodos:**

Estudo qualitativo por abordagem estrutural das representações sociais realizada em um serviço de cirurgia, Brasil. Amostra de seleção completa (n=180) com delineamento temporal composta por mulheres (idade ≥18 anos) submetidas à punção venosa durante procedimentos anestésico- cirúrgicos. Utilizou-se técnica de evocação livre de palavras com o termo indutor “pegar veia para anestesia e cirurgia”. Realizou- se análise prototípica empregando o *software* EVOC2003. Os requisitos ético-legais foram atendidos.

**Resultados:**

As mulheres tinham de 20 a 39 anos (63%) e 10 a 13 anos de escolaridade (56,1%). No possível núcleo central constam sentimentos (“dor” e “medo-sem-medo”) em oposição à área de contraste “(in)certeza- habilidade-profissional” e “tranquila-fácil” e com elementos objetivados superativados alocados na primeira periferia, retratando estressores intrapessoais, interpessoais e extrapessoais.

**Discussões:**

A dor, enquanto objeto representacional, remeteu à função justificadora quando aproximada da agulha, reafirmando o surgimento de medo, nervosismo e da ansiedade (estressores).

**Conclusões:**

Dor, medo, ansiedade e nervosismo foram estressores identificados nas representações sociais que necessitam ser monitorados terapeuticamente por meio de relações interpessoais de confiança entre profissional-usuário, aliando a performance do desempenho, sendo necessário inserir a educação permanente para profissionais que puncionam vasos.

## Abstract (en)

**Introduction:**

Vein catheterization is essential for anesthetic and surgical procedures as its characteristics are compatible with stability, flow volume in short periods of time and thick caliber to avoid vascular trauma.

**Objective:**

To identify women’s social representations of vein puncture for anesthetic and surgical purposes in the light of Neuman’s stressors.

**Materials and Methods:**

A qualitative study with structural focus on social representations was conducted in a medical surgical unit in Brazil. A complete selection sample (n=180) made up of women (age ≥ 18 years) undergoing vein puncture for anesthetic and surgical procedures was carried out. The free lexical retrieval technique was used with “vein puncture for anesthesia and surgery” as inductive term. A prototypical analysis was performed using EVOC 2003 software. Ethical and legal standards were also met.

**Results:**

63% were women aged 20 to 39 years and 56.1% had 10 to 13 years of schooling. In the possible nuclear element, feelings (“pain” and “fear”) were found opposed to the contrast zone “(in)certainty-professional skills” and “calm-tranquility” with overactivated objective elements allocated in the first periphery representing intrapersonal, interpersonal and extrapersonal stressors.

**Discussion:**

Pain, as an object of representation, referred to the justifying function when approached by the needle, which reaffirms the presence of fear, nervousness and anxiety (stressors.)

**Conclusions:**

Pain, medication, anxiety and nervousness were identified in social representations, which need to be therapeutically controlled through interpersonal relationships of trust between professionals and users, in addition to vein puncture performance, for which it is necessary to promote continuing education among professionals.

## Resumen

**Introducción:**

El cateterismo venoso es esencial para los procedimientos anestésicos y quirúrgicos, cuyas características son compatibles con la estabilidad, el volumen de flujo en periodos cortos de tiempo y el grueso calibre para evitar el trauma vascular.

**Objetivo:**

Identificar las representaciones sociales de las mujeres sobre la punción venosa con fines anestésicos y quirúrgicos a la luz de los estresores de Neuman.

**Materiales y métodos:**

Estudio cualitativo con enfoque estructural en las representaciones sociales realizado en una unidad de cirugía en Brasil. Se realizó una muestra de selección completa (n=180) con delimitación temporal compuesta por mujeres (edad ≥ 18 años) sometidas a venopunción durante procedimientos anestésicos y quirúrgicos. Se utilizó la técnica de evocación lexical libre con el término inductivo “toma de vena para anestesia y cirugía” y se realizó un análisis prototípico mediante el software EVOC 2003. Asimismo, se cumplieron los requisitos ético-legales.

**Resultados:**

Las mujeres tenían entre 20 y 39 años (63%) con 10 y 13 años de escolaridad (56.1%). En el posible elemento nuclear, se encuentran sentimientos (“dolor” y “miedo”) en oposición a la zona de contraste “(in)certidumbre- habilidad profesional” y “calma-tranquilidad”, con elementos objetivos sobreactivados asignados en la primera periferia que representan estresores intrapersonales, interpersonales y extrapersonales.

**Discusión:**

El dolor, como objeto de representación, remitió a la función justificadora cuando se les acercó la aguja, lo que reafirma la aparición del miedo, el nerviosismo y la ansiedad (estresores).

**Conclusiones:**

En las representaciones sociales se identificó al dolor, la medicación, la ansiedad y el nerviosismo como factores estresantes, los cuales necesitan ser controlados terapéuticamente mediante relaciones interpersonales de confianza entre el profesional y el usuario, además del desempeño en la punción, para lo que es necesario promover la educación continua de los profesionales encargados de esta tarea.

## Introdução

Tendo em vista a identificação das mulheres como maioria da população brasileira (50,77%) e principais usuárias do Sistema Único de Saúde (SUS), é necessário refletir sobre as políticas nacionais de saúde da mulher. No Brasil, nas primeiras décadas do século XX, as políticas limitavam-se às demandas relativas à gravidez e ao parto, tendo sido incluídas posteriormente ações educativas, preventivas, de diagnóstico, tratamento e recuperação da saúde, englobando a assistência à mulher em clínica ginecológica, no pré-natal, parto e puerpério, no climatério, em planejamento familiar, infecções sexualmente transmissíveis (ISTs), câncer de colo de útero e de mama^1^.

Apesar dos avanços, identificam-se lacunas na atenção ao climatério/menopausa; queixas ginecológicas; infertilidade e reprodução assistida; saúde da mulher na adolescência; doenças crônico-degenerativas; saúde ocupacional; saúde mental; doenças infectocontagiosas e a inclusão da perspectiva de gênero e raça nas ações a serem desenvolvidas^1^.

Frente a essa ampliação da atenção à saúde das mulheres, decorrente também da feminilização do envelhecimento, verificamos a predominância delas também na prática anestésica para procedimentos cirúrgicos (64,8%) associada principalmente às doenças crônico-degenerativas prevalentes no envelhecimento, a cirurgias gerais ou às de especialidade ginecológica^2^.

Para implementar o atendimento anestésico-cirúrgico, a realizaçãodapunçãovenosaéessencial. Apunçãovenosa, realizada cotidianamente nas instituições hospitalares, envolve múltiplos fatores como: habilidades técnicas e comunicacionais; tecnologia de equipamentos e insumos; e a percepção do usuário sobre a necessidade do procedimento e de como ele é realizado, acessado por meio do surgimento de manifestações físicas (trauma vascular periférico), emocionais (crenças e vivências anteriores) e psicológicas (medo e ansiedade) que constituem em estressores para as pessoas submetidas ao processo de punção venosa periférica. O processo de punção venosa periférica está sendo concebido como o procedimento técnico, comunicacional, interpessoal e terapêutico realizado por profissionais de saúde com habilidades relacionais e técnicas capazes não só de introduzir, manter, remover e prestar cuidados pós-remoção de um cateter venoso periférico, mas também modular os possíveis estressores que surgirem durante sua utilização para intervenções anestésico-cirúrgicas.

A fonte de estressores em mulheres submetidas à punção venosa precisa ser identificada para que práticas direcionadas a elas sejam aplicadas para uma assistência humanizada e segura. Os estressores são componentes que necessitam ser apreendidos na perspectiva da pessoa cuidada, por constituírem em fatores intervenientes sobre a percepção do cuidado profissional recebido, condição descrita anteriormente em estudos que o abordaram o processo de punção venosa na perspectiva do usuário e dos acompanhantes^3^^,^^4^, por influenciar na percepção de qualificação profissional, segurança e bem-estar do usuário, e no êxito terapêutico.

No presente estudo os estressores estão sendo concebidos como a reunião de forças de origem intrapessoal, interpessoal e extrapessoal que se manifestam por meio das variáveis biológicas, psicológicas, socioculturais, desenvolvimentais e espirituais afetando as linhas de defesa flexível, normal e de resistência que constituem em recursos de energia da estrutura básica que variam de intensidade e tamanho segundo o indivíduo^5^. Nesse sentido, a Teoria dos Sistemas de Betty Neuman^5^, como modelo teórico-filosófico, aliada à Teoria das Representações Sociais, possibilita identificar conhecimentos, imagens, valores, crenças, percepções, sentimentos, atitudes e comportamentos^6^ que retratam possíveis estressores intra, inter e extrapessoais^5^ durante a punção venosa para fins de procedimentos anestésico-cirúrgicos em mulheres. Pode- se assim refletir sobre as formas de enfrentamento das representações compartilhadas pelo grupo social, sendo, portanto, delineadas como um objeto representacional.

Acredita-se que as mulheres que têm seus vasos puncionados para fins anestésico-cirúrgicos constituam um grupo socialmente contextualizado, podendo acessar objetos representacionais passíveis de ser captados pelo “senso comum”^7^.

A justificativa desta investigação está alicerçada na prevalência de mulheres nos procedimentos anestésico-cirúrgicos e por ser a punção venosa periférica (PVP) necessária para viabilizá-los. Desse modo, os estressores representados para PVP em mulheres submetidas a procedimento anestésico-cirúrgico foram objeto desta investigação.

Diante do exposto, objetivou-se discutir as representações sociais de mulheres sobre punção venosa para fins anestésico-cirúrgicos à luz dos estressores de Neuman.

## Materiais e Métodos

Pesquisa qualitativa delineada na abordagem estrutural da Teoria das Representações Sociais (TRS)^6^^,^^7^, apresentada segundo protocolo *Consolidated criteria for reporting qualitative resarch* (COREQ)^8^ e analisada com alicerce dos estressores da Teoria dos Sistemas de Betty Neuman^5^. Foi cenário da investigação um serviço de cirurgia de um hospital de ensino filantrópico com leitos conveniados ao Sistema Único de Saúde (SUS) em Minas Gerais, Brasil. Trata-se de hospital geral com maternidade de referência (pré-natal e parto de alto risco) de uma macrorregião com aproximadamente dois milhões de usuários.

A amostra foi de seleção completa com recorte temporal (coleta de dados foi realizada no período de julho/2019 a janeiro/2020), cujos 180 participantes foram recrutados por convite individual no período cirúrgico.

O número de participantes foi definido com base nas recomendações para estudos em TRS na abordagem estrutural (n= população total), sendo abordado o número total de mulheres (n = 191) submetidas a punção venosa para fins anestésico-cirúrgicos no setor cirúrgico^9^.

Foram critérios de elegibilidade: ser mulher com idade ≥18 anos, ter pelo menos uma veia periférica puncionada para fins anestésico-cirúrgicos e estar internada no setor cirúrgico. Foram excluídas mulheres que apresentaram intercorrências pós-cirúrgicas ou tiveram relato de dor ou desconforto a ponto de inviabilizar sua participação ou aquelas que tiveram interrupção na entrevista, inviabilizando sua finalização. Houve 11 perdas, sendo seis por intercorrência pós- cirúrgica; quatro que relataram dor/desconforto a ponto de inviabilizar sua participação e uma devido à interrupção na coleta que inviabilizou sua conclusão, delimitando a amostra em 180 participantes.

O pesquisador (sexo masculino) responsável pela obtenção dos dados é membro da equipe de saúde da instituição na qual se deu a coleta de dados, o que favoreceu sua inserção no cenário e minimizou interferências na rotina cotidiana do serviço. Para minimizar risco de exposição das participantes, foi utilizado tom de voz adequado ao diálogo e biombos, visto que as entrevistas se deram em leitos situados em espaços onde havia mais de uma paciente. O instrumento de coleta de dados foi estruturado em: 1) caracterização sociodemográfica; 2) entrevista individual com aplicação da técnica de evocação livre de palavras (TALP) desencadeada pelo termo indutor “pegar veia para anestesia e cirurgia”.

A TALP foi utilizada como técnica de evocação para acessar os conteúdos representacionais das participantes. Assim, foi solicitado a cada participante que mencionasse as primeiras cinco palavras que lhe viessem à mente quando o termo indutor foi mencionado. Esta etapa metodológica foi realizada por meio de entrevista individual e os cognemas evocados foram registrados na ordem de menção. A TALP consiste em uma técnica projetiva que busca evidenciar conteúdos latentes não filtrados pela censura. Nesse sentido, não houve devolutiva dos conteúdos da entrevista para as participantes.

A coleta de dados foi realizada no período de julho/2019 a janeiro/2020. A aplicação do instrumento teve duração média de 10 minutos e foi realizada à beira do leito, na sala de recuperação pós-anestésica ou na unidade de internação cirúrgica. Os dados foram registrados à beira do leito em formulário eletrônico em dispositivo android com suporte do aplicativo *Open Data Kit*.

As variáveis quantitativas de caracterização dos participantes foram consolidadas no S*oftware Statistical Package for Social Science for Windows* (SPSS) versão 24, sendo analisadas segundo estatística descritiva, medidas de dispersão e tendência central.

Os cognemas evocados foram tratados pela técnica do dicionário, que consiste em agrupar palavras/expressões semelhantes segundo critérios lexicais e semânticos, excluindo artigos, preposições e fazendo aproximações de expressões sínteses com hífem^10^. O *corpus* de evocações foi tratado no programa EVOC 2003 por análise prototípica, gerando o quadro de quatro casas. Foram evocadas 812 palavras-expressões por 180 mulheres, em que foram identificadas 65 palavras-expressões distintas. Adotou-se como pontos de corte para a composição da análise prototípica: frequência mínima de 21, frequência intermediária de 90 e *rang* médio de 2,6. Foram utilizados 30,5% do *corpus* dos cognemas (expressões ou palavras evocadas) a partir da aplicação do critério da Lei de Zipf^11^.

Todos os requisitos ético-legais de pesquisa em humanos foram atendidos. Às participantes foram esclarecidos os objetivos do estudo e os procedimentos de coleta de dados. Àquelas que concordaram em participar da investigação foi solicitado a assinatura do termo de consentimento livre e esclarecido antes da coleta dos dados. Para assegurar o anonimato das participantes, foram atribuídos códigos contendo cinco dígitos alfanuméricos, sendo sequenciais, duas letras e três algarismos para fins de identificação.

Cabe mencionar que este manuscrito integrou uma investigação matriz intitulada: “Processo de punção de vasos periféricos e trauma vascular: estudo de intervenções complexas usando método misto em centro cirúrgico” (Parecer n° 3.198.431, aprovado em 14 março/2019- Comitê de ética da Universidade Federal de Juiz de Fora).

## Resultados

As 180 participantes ficaram assim caracterizadas: 67,2% tinham idade menor que 40 anos (33± 10,926; 18-73 anos); 54,1% tinham mais de dez anos de escolaridade (13± 3,020; 0-18); 63,5% recebiam de um a dois salários mínimos (1,00±0,888; 0-5); 60,3% tinham filhos (2± 1,126; 0-5); 32,8% passaram por partos vaginais (0,00±0,979;0-4); 99,5% tinham passado por pelo menos um procedimento cirúrgico prévio à atual internação (1,00±0,762;1-5); 23,8% tinham pelo menos uma doença prévia conhecida (0,00±0,611;0-3). O tempo de internação variou de um a cinco dias.

**Figura 1:**
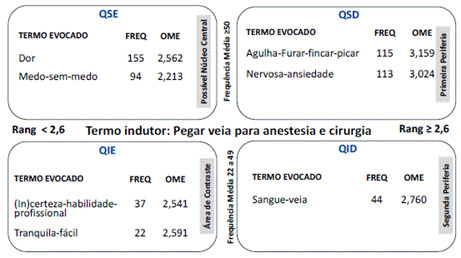
Quadro de quatro casas para o termo indutor “pegar veia para anestesia e cirurgia”. Juiz de Fora (MG), 2019-2020.

Na análise prototípica, foi possível obter o quadro de quatro casas (Figura 1).

Fonte: Dados da pesquisa. Nota: conteúdos extraídos do Programa Evoc2003.

No quadro de quatro casas (Figura 1), a representação social (RS) resultante dos termos evocados pelas mulheres submetidas à punção venosa para fins anestésico-cirúrgicos foi objetivada pelo sentimento de dor e de medo (possível núcleo central).

Essa RS é ancorada por cognemas que expressam a valoração da habilidade profissional (área de contraste), pelo objeto utilizado para puncionar - a agulha, pela atitude profissional de “furar” a veia, pelo sentimento de ansiedade/nervosismo (primeira periferia) e pela presença de sangue (segunda periferia) que caracterizam, reforçam, dão sentido e integram o sistema de valores do grupo social.

O termo valorativo tranquilo/fácil (área de contraste) expressa o termo menos evocado representando uma oposição ao senso comum compartilhado pelas mulheres. Neste quadrante é apresentada posição oposta de algumas mulheres na forma pela qual enfrentam o procedimento de punção venosa para fins anestésico-cirúgicos.

A aproximação dos conteúdos representacionais com os estressores de Neuman foi avaliada por cada um dos pesquisadores e obtido consenso. Os conteúdos das dimensões representacionais derivaram dos dados organizados no quadro de quatro casas; e ao serem aproximados dos estressores de Neuman, possibilitaram identificar comportamentos, atitudes, sentimentos, conhecimentos, informações, valores e objetos. Estes representaram formas de enfrentamento do grupo social de mulheres submetidas a procedimentos anestésico- cirúrgicos que retrataram estressores quando submetidas à punção de veias periféricas. Esses estressores foram: 1) intrapessoais: dor, medo e ansiedade e sangue- veia; 2) interpessoais: (in)certeza quanto à habilidade profissional; e 3) extrapessoais: agulha que era utilizada para “furar-fincar-picar”.

## Discussão

Pelo fato de o cenário da investigação conciliar a condição de ser uma instituição hospitalar geral e maternidade de referência (pré-natal e parto de alto risco), o perfil das participantes foi semelhante ao de gestantes de alto risco apresentado em outro estudo^12^.

A representação de se puncionar veia para fins anestésico-cirúrgicos foi vinculada a dor e medo, elementos mais frequentemente mencionados e com maior prontidão. Na análise prototípica estão alocados no quadrante superior esquerdo (QSE). Apesar do fato de se ter considerado apenas mulheres nesta investigação, foi possível identificar uma aproximação com a RS sobre punção de vasos na perspectiva de homens e mulheres hospitalizados^3^. Esses comportamentos, atitudes e sentimentos podem ser justificados pela necessidade de o profissional anestesista dispor de uma veia compatível com a introdução de cateter intravascular (CIV) de calibre adequado à infusão de fluidos e medicamentos para viabilizar o procedimento cirúrgico^13^^,^^14^.

A finalidade da punção venosa periférica utilizada inclui assegurar a estabilidade/constância da posição do CIV durante todo o período transoperatório (ato anestésico e cirúrgico) até o período do pós-operatório imediato (retorno ao nível de consciência e sensibilidade com estabilidade hemodinâmica) e possibilitar a infusão de altos volumes em curtos espaços de tempo em caso de instabilidade hemodinâmica ou intercorrência^13^^,^^15^^-^^17^.

O surgimento da dor justifica-se pelo rompimento de estruturas corporais pelo dispositivo agulhado e pela proximidade do calibre do CIV com o diâmetro do vaso. Na medida em que há o contato/atrito do cateter agulhado com a íntima do vaso ainda há a possibilidade de desencadear flebite de origem mecânica, o que também implica em experiência dolorosa^13^^,^^16^^,^^18^. Entretanto, o procedimento anestésico, motivador da realização da punção intravascular, possui, em sua essência, a meta de controlar e reduzir a dor advinda do ato cirúrgico. Visa garantir a viabilidade de acesso a infusões com efeito rápido e promover o controle de princípios ativos e volumétricos em casos de instabilidade hemodinâmica^19^.

Embora o medo seja componente descrito na literatura como elemento representacional vinculado a punção de vasos periféricos em outros grupos sociaise^3^^,^^20^ e faixas etárias^21^, é possível que ele, no caso das punções para fins anestésico-cirúrgicos, possa se confundir com o medo/a insegurança da anestesia e do próprio procedimento cirúrgico. Em outro estudo, do qual 84,6% dos participantes eram mulheres, a ansiedade no pré- operatório foi considerada fator preditivo para dor no pós-operatório^22^.

No quadrante inferior esquerdo (QIE), foram alocados dois cognemas: “tranquila-fácil” e “(in) certeza-habilidade-profissional”. A expressão “tranquila-fácil” constitui um componente valorativo que se refere ao comportamento de resiliência dos sujeitos sociais diante da punção de um vaso. Ele retrata o oposto da forma como as mulheres lidaram majoritariamente com a punção de vasos periféricos, expressa pelos cognemas “medo-sem-medo” e “dor” alocados no QSE.

A incerteza/certeza quanto à habilidade do profissional para a realização do procedimento pode remeter à consolidação da imagem da punção de vasos vinculada às atividades realizadas pela enfermagem. Isso porque, embora a realização de punção de vasos periféricos seja uma atividade laboral cotidiana da prática de enfermeiros^13^^,^^14^^,^^16^, na presente investigação, pelo fato de sua realização ocorrer para fins anestésico-cirúrgicos, ela foi operacionalizada por médicos anestesistas. Tal fato constitui um componente representacional não descrito anteriormente na literatura.

Em um estudo que utilizou a abordagem estrutural da TRS e que foi realizado com pais/ acompanhantes de crianças internadas sobre a punção de vasos periféricos de seus filhos/ tutelados, cujo procedimento foi operacionalizado pela equipe de enfermagem, a habilidade profissional não constituiu componente representacional^22^ fato corroborado em outra investigação conduzida com adultos em situações clínico-cirúrgicas^21^.

Cabe mencionar que o fato de o cognema “(in)certeza-habilidade-profissional” estar alocado no QIE remete à flexibilidade e à acomodação necessária para que tenha a função de proteção do núcleo central, justificando a presença dos elementos alocados no QSE. Isso equivale a dizer que a função estabilizadora do núcleo central é fundamental para a constância de uma representação social^6^^,^^7^.

A construção dos vínculos entre profissional e usuário pode ficar comprometida e justificar a ocorrência de ansiedade, medo e insegurança, tendo em vista que a visita do anestesista ao paciente ocorre, nos casos em que o período de internação é curto, no dia do procedimento cirúrgico ou na véspera do mesmo, e seu foco está centralizado na avaliação dos exames pré- anestésicos e no esclarecimento de dúvidas^23^. Cabe acrescentar que outro fator interveniente é a aparência do profissional no encontro subsequente, uma vez que, nessa ocasião, ele estará com vestes padronizadas e usando gorro e máscara, o que dificulta para o paciente a identificação fisionômica do profissional com quem teve contato no encontro anterior^24^.

No caso do cognema que remete à (in) segurança da ação profissional, ele se liga à inexistência de vínculos da díade profissional-paciente que sejam capazes de promover estabilidade emocional às mulheres que passam por procedimentos técnicos como o da punção de vasos periféricos^25^^,^^26^.

No quadrante superior direito (QSD) emergiram duas expressões: “agulha-furar-fincar-picar” e “nervosa-ansiedade”, que podem ser consideradas elementos periféricos superativados em virtude da frequência com que foram mencionados e pelo fato de seus valores se aproximarem da frequência dos componentes alocados no possível núcleo central, sendo a ordem média de evocação a condição que normatiza a alocação desses elementos na primeira periferia^27^^,^^28^.

A“agulha” foi o componente ligado ao termo indutor e considerada a fonte do desconforto (dor), a geradora do sentimento de medo, nervosismo e ansiedade, vinculada à ação que exerce, ou seja, o ato de “furar”, “fincar” e “picar”. A “agulha” também foi objeto central da representação de pais/tutores quando as crianças passaram pela experiência de punção de vasos periféricos por ocasião do período de internação hospitalar^21^.

Na segunda periferia, QID, está alocada outra expressão objetivada: “sangue-veia”. Trata-se de componentes reificados, uma vez que o sangue, ao retornar para o interior do cateter, constitui marcador do momento em que a agulha alcançou o interior da veia e possibilita comprovar o êxito do procedimento pelo profissional^13^^,^^14^^,^^16^. Na presente investigação, esses parâmetros também foram mencionados pelas participantes que tiveram seus vasos puncionados.

Embora o retorno do fluxo sanguíneo seja marcador do êxito da entrada da agulha no interior do vaso, a utilização de tecnologias de imagem é recomendada para melhorar a assertividade do procedimento de punção de vasos periféricos. Mostram-se compatíveis do ponto de vista do custo-benefício, além de reduzir a ansiedade das pessoas que se sentem inseguras ou possuem veias de difícil punção^29^. Em uma investigação com 150 pessoas adultas internadas para tratamento clínico-cirúrgico, a ansiedade e o nervosismo foram identificados no núcleo central da TRS^3^.

Ao analisar o surgimento de estressores de origem intrapessoal, interpessoal e extrapessoal na perspectiva de mulheres submetidas ao processo de punção vasos periféricos para fins anestésico-cirúrgicos à luz da do cuidado individualizado e humanizado, faz-se necessário implementar ações terapêuticas que sejam capazes de remodelar tais estressores e restabelecer o equilíbrio das linhas energéticas de defesa flexível, normal e de resistência, quando afetadas, favorecendo a vivência de pessoas que necessitam ter seus vasos periféricos puncionados, à semelhança de resultados obtidos em outras investigações^3^^,^^4^.

O processo sociocognitivo da objetivação refere-se à forma como são organizados os elementos que constituem as representações e o percurso através do qual tais elementos adquirem materialidade e se tornam expressões de uma realidade pensada como natural. Ele se desenvolve por meio de uma construção seletiva das informações sobre o objeto, esquematização para organização dos elementos e naturalização, em que os conceitos obtidos passam a se constituir como categorias naturais e materiais, de modo que estudar as relações entre os objetos de uma representação é estudar a sua objetivação^30^.

A ancoragem refere-se à assimilação de um objeto novo por objetos já presentes no sistema cognitivo. Esses objetos são as “âncoras” que permitem construir a representação do novo objeto e sustentá-la. O estudo da ancoragem analisa a relação entre as pertenças sociais e os conteúdos de uma representação, a partir da hipótese de que as experiências comuns aos membros de um mesmo grupo, decorrentes de uma mesma inserção no campo das relações sociais, suscitam representações semelhantes^30^.

A RS da punção venosa para fins anestésico-cirúrgicos por mulheres ao reproduzir os significados distintos dos sujeitos sobre o objeto, trazer aquilo que é abstrato, a imagem mental que possuem sobre o mesmo, e o conceito da imagem, enquanto processo de elaboração dos conhecimentos do grupo social a respeito do objeto/imagem foi objetivada pelo sentimento de dor e de medo. A ancoragem, ao retratar o meio pelo qual a RS se enraíza nas relações sociais e nos valores dos sujeitos descreve a relação entre os cognemas da RS, as categorias que justificam e dão sustentação aos conteúdos alocados no núcleo central pela posição em que os elementos periféricos se apresentam, ancorando a RS na realidade dos sujeitos pelos objetos utilizados para puncionar a exemplo da agulha, da presença de sangue, da atitude profissional necessária de “furar” a veia culminando em sentimentos de ansiedade/nervosismo com função justificadora e normativa do núcleo central da RS^30^^,^^31^.

A dor foi ancorada no objeto da representação, remeteu à função justificadora quando aproximada da agulha, à função normativa diante da presença do sangue na veia e possibilitou justificar o surgimento de medo, nervosismo e ansiedade. A função funcional não foi mencionada, uma vez que o foco da representação foi influenciado pelo procedimento cirúrgico, o que fez com que a punção do vaso se mostrasse complementar no processo representacional.

Na presente investigação, foram identificados três tipos de estressores: 1) intrapessoal: representado por dor, medo, ansiedade e nervosismo; 2) interpessoal: retratado pelo questionamento quanto à habilidade profissional para a realização do procedimento de punção e expresso pelo cognemas (in)certeza-habilidade profissional; e 3) extrapessoal: manifestado pela presença de sangue-veia, da agulha e sua ação, ou seja, “agulha-furar-fincar-picar”.

Os estressores decorrem de fluxos de energia que atuam sobre o sistema energético de defesa flexível, normal e sobre as linhas de resistência, impactando o *continuum* saúde-doença e causando instabilidade nele. Seu impacto pode ser ou não percebido pela pessoa, requerendo uma intervenção em nível primário, a qual visa à reestabilização do sistema de forças. Quando se manifesta com percepção e reações por parte da pessoa, ela requer intervenções de nível secundário que podem ser efetivadas por meio da reestruturação energética das linhas de defesa normal e flexível. Na ocasião em que as linhas de resistência são afetadas, há comprometimento de todo o sistema, colocando em risco a vida^5^.

Quando se almeja uma assistência de qualidade e a segurança do paciente, há necessidade de serem monitorados terapeuticamente esses estressores e implementar intervenções terapêuticas preventivas. Uma das estratégias passíveis de ser implementadas é construir relações interpessoais de qualidade, baseadas na confiança entre profissional-paciente. Aliado à habilidade comunicacional e relacional, é possível melhorar o desempenho dos profissionais por meio de processos de educação permanente e agregar a utilização de tecnologia apropriada à melhoria da visualização dos vasos^4^^,^^32^.

A presente investigação traz como contribuições para a enfermagem a necessidade de reavaliar e ressignificar o processo de punção de vasos para fins cirúrgico-anestésicos, considerando que esse procedimento desencadeia estressores intrapessoais, interpessoais e extrapessoais apontando para intervenções capazes de interferir na experiência dolorosa, no medo e na ansiedade diante da vivência de ter o vaso puncionado.

O fato de os dados remeterem às mulheres que foram submetidas a procedimentos anestésico- cirúrgicos no contexto do estudo impede a generalização dos resultados para outros grupos sociais, sendo uma limitação deste estudo. Portanto, sugere-se a realização de estudos utilizando a abordagem processual para dar voz aos sujeitos coletivos e aprofundar a origem de tais representações.

## Conclusões

Os constructos simbólicos representacionais sobre como a RS de mulheres que vivenciam a punção de vasos periféricos para fins anestésico-cirúrgicos são objetivados nos sentimentos dor e medo evidenciados nos estressores intrapessoais (dor, medo e ansiedade); interpessoais advindos da (in)certeza-da habilidade do profissional e extrapessoais com o uso de agulha utilizada para “furar-fincar-picar” e do sangue e da veia.

Para haver a redução dos estressores, é necessário consolidar as relações interpessoais entre profissionais que puncionam vasos e a pessoa que tem seu vaso puncionado, visando à construção de confiança, além de garantir que os profissionais disponham de educação permanente sobre as relações interpessoais, vinculando-as com o desempenho da técnica de punção venosa periférica. Considerando que o processo de punção de vasos periféricos é uma prática realizada no cotidiano dos profissionais da equipe de enfermagem, os resultados da presente investigação alertam tais profissionais para as respostas humanas identificadas nesta investigação que se mostram compatíveis com intervenções terapêuticas de enfermagem e processos de reconstituição das linhas energéticas daqueles que têm seus vasos puncionados alicerçadas em uma relação em que o cuidado é empático, humanizado e de excelência técnica.
